# Coinfection ecology and pathogen emergence in a *Borrelia*-endemic landscape: 5 years of *Borrelia burgdorferi*, *Anaplasma phagocytophilum*, and *Babesia microti* surveillance in Maryland

**DOI:** 10.1128/aem.02242-25

**Published:** 2026-04-20

**Authors:** Greg Joyner, Olifan Abil, Maria J. Sanches, Amy Schwartz, Julia Poje, Kathryn Arnold, Christine Petersen, Maria Gomes-Solecki

**Affiliations:** 1 Department of Microbiology, Immunology and Biochemistry, University of Tennessee Health Science Center 274062, Memphis, Tennessee, USA; 2 Immuno Technologies Inc., Memphis, Tennessee, USA; 3 University of Iowa 4083, Iowa City, Iowa, USA; Universite de la Reunion, Ste Clotilde, France

**Keywords:** *Borrelia burgdorferi*, *Peromyscus leucopus*, *Ixodes scapularis*, ecology, coinfection, *Anaplasma phagocytophilum*, *Babesia microti*, pathogen emergence, enzootic transmission, tick-borne

## Abstract

The emergence of tick-borne pathogens depends on ecological opportunity and barriers to persistence within vectors and hosts. *Borrelia burgdorferi* is well established in the mid-Atlantic, whereas *Babesia microti* and *Anaplasma phagocytophilum* remain patchily distributed. Five years of integrated surveillance (2020–2024) at three Maryland sites allowed us to track *B. microti* and *A. phagocytophilum* establishment by screening questing *Ixodes scapularis* nymphs, *Peromyscus leucopus-*fed nymphs, and *P. leucopus* samples by qPCR, contextualized with county-level human case data. *B. burgdorferi* was consistently detected in all sites and sample types, with prevalence generally 5%–20% in questing nymphs and exceeding 30% in hosts, confirming long-term enzootic maintenance. By contrast, *B. microti* and *A. phagocytophilum* were initially sporadic but increased in rodents and *P. leucopus-*fed ticks. Over time, *A. phagocytophilum* prevalence significantly increased to above 20% in some *P. leucopus-*fed nymphal collections despite much lower prevalence in questing ticks, highlighting the early-warning value of blood meal-associated surveillance. Notably, *B. microti* reached very high prevalence in *P. leucopus* hosts at a specific site (up to ~80%) while remaining rare or absent in questing and engorged nymphs, highlighting a pronounced decoupling between host infection and vector prevalence. Coinfections were rare, though enrichment of *B. burgdorferi + A. phagocytophilum* in *P. leucopus-*fed ticks suggests possible facilitation during early establishment. These results indicate that *B. microti* and *A. phagocytophilum* are actively emerging in Maryland, following their establishment in the Northeast and Upper Midwest. Combining surveillance from questing nymphs, *P. leucopus-*fed nymphs, and reservoir hosts provides a framework for detecting enzootic cycles before they appear in questing ticks or human case counts, offering early-warning capacity for public health preparedness.

**IMPORTANCE:**

Understanding why some tick-borne pathogens become ecologically established while others remain sporadic is central to predicting human disease emergence. By combining surveillance of questing nymphal *Ixodes scapularis* ticks, *Peromyscus leucopus-*fed nymphal ticks, and *Peromyscus leucopus* reservoir hosts across 5 years in Maryland, we show that *Babesia microti* and *Anaplasma phagocytophilum* remain in the early stages of enzootic establishment, whereas *Borrelia burgdorferi* is deeply established. This integrated approach demonstrates how pathogen biology within the tick shapes field prevalence and highlights *P. leucopus-*fed ticks as a powerful xenodiagnostic early-warning tool for detecting emerging pathogens before they are reflected in questing populations or human case data.

## INTRODUCTION

Lyme disease, caused by *Borrelia burgdorferi*, is deeply ecologically established in the northeastern and mid-Atlantic United States. In contrast, *Babesia microti* (babesiosis) and *Anaplasma phagocytophilum* (anaplasmosis) have more recently emerged as enzootic cycles in these regions. The broader category of tick-borne diseases continues to expand its geographic and public health impact (1, 2), with *A. phagocytophilum* and *B. microti* posing new challenges for surveillance and control due to their complex ecology, varied transmission dynamics, and potential for asymptomatic infection in humans (3–8).

The enzootic cycle of *B. burgdorferi* is characterized by high reservoir competence in *Peromyscus leucopus*, efficient acquisition by *Ixodes scapularis*, and consistent vector-to-host transmission (9–12). In contrast, *B. microti* and *A. phagocytophilum* display distinct patterns of prevalence, persistence, and transmission efficiency, particularly in emerging regions (13, 14). *Babesia microti* is a protozoan parasite with a two-host life cycle involving obligate vertebrate and arthropod stages. Infection in rodents is typically asymptomatic and can persist for months, providing a sustained source of transmission to feeding larvae (5, 15). Ticks must acquire *B. microti* as larvae to be infectious as nymphs, as transstadial passage is required and transovarial transmission does not occur (11, 12).

*A. phagocytophilum* is an obligate intracellular bacterium that replicates within neutrophils in vertebrate hosts and within midgut or salivary tissues in the tick vector. Enzootic maintenance depends primarily on larval acquisition, with nymphs serving as the main transmission stage; thus, most nymphal infections reflect prior larval acquisition (6, 10, 16, 17). However, nymphal acquisition is biologically possible, though rarely demonstrated experimentally, and would generate infected adults, which may contribute to human risk even if this pathway plays little role in long-term persistence. Field detections of *A. phagocytophilum* in adult *I. scapularis* have been observed (18, 19). Transovarial transmission has not been demonstrated (17, 20, 21).

Coinfections involving *B. burgdorferi* and *B. microti* are increasingly recognized in clinical settings and may contribute to more severe or prolonged illness in immunocompromised patients (22–26). In contrast, coinfections with *A. phagocytophilum* are less consistently associated with exacerbated clinical outcomes, and current evidence does not suggest a clear augmentative effect between *A. phagocytophilum* and other tick-borne pathogens (27). Nonetheless, such coinfections remain diagnostically important, especially in transitional regions where ecological signals may precede clinical awareness.

At the ecological level, coinfection of *Ixodes scapularis* with multiple pathogens has been documented across endemic and transitional zones, though mechanisms remain incompletely understood. Shared hosts and synchronized seasonal dynamics may contribute to coinfection, but there is also speculation that the presence of one pathogen might facilitate acquisition or persistence of another. For example, *B. microti* prevalence in ticks often lags but tracks that of *B. burgdorferi*, potentially reflecting the use of established host–vector systems during regional expansion (28–32).

Rather than seeking to establish first detection, this study aims to describe the trajectory of *B. microti* and *A. phagocytophilum* emergence in Maryland, where establishment appears to be ongoing. To avoid ambiguity, we use the following operational definitions throughout. We use sporadic detection for isolated detections without evidence of persistence across years and/or across both ticks and reservoir hosts. We use emergence for patterns consistent with a developing enzootic cycle, such as increasing detection frequency over time and/or broader spatial occurrence, particularly when accompanied by detections in reservoir hosts and their associated ticks. We use established (enzootic maintenance) for sustained transmission supported by repeated detections across multiple years together with detections in hosts and host-fed ticks, consistent with a self-maintaining cycle in this system. We use endemic for long-standing, established transmission with relatively stable occurrence over time. Our approach builds on previous calls for long-term, integrated surveillance across both hosts and vectors to better understand interannual dynamics (2, 4, 9, 33).

Historical studies in endemic regions such as southern New York and Nantucket Island demonstrated stable cycles of *B. microti* in rodents and nymphs decades ago, yet similar establishment only recently extended further south, often after decades of lag relative to *B. burgdorferi* (18). Surveillance of blood donors and hospitalized patients has revealed increasing cases of babesiosis in previously low-incidence states, including a >1,400% rise in case numbers in Maine and Vermont between 2011 and 2019 (34). A recently published study evaluating *Ixodes scapularis* across the eastern seaboard indicated the emergence of *Babesia microti* in Maryland and Delaware between 2013 and 2023, also finding coinfected ticks (35). Maryland lies at a critical latitudinal and ecological transition between historically endemic zones (e.g., New York, Pennsylvania) and regions where *B. microti* and *A. phagocytophilum* have only recently become detectable. Its climatic convergence with the Northeast, along with forest fragmentation and dense *Peromyscus* spp. populations, makes it an ideal sentinel zone for tracking ecological penetrance (31, 36).

We present a 5-year data set (2020–2024) from three geographically separated sites in Maryland (Fig. S1), each sampled consistently using drag and live-trapping protocols and analyzed via qPCR for *B. burgdorferi*, *B. microti*, and *A. phagocytophilum*. Infection data from questing nymphs, host-attached nymphs, and *Peromyscus leucopus* hosts are synthesized alongside county-level human case reports to assess the degree of establishment. While coinfections were infrequent in this data set, they remain epidemiologically relevant due to their potential to exacerbate disease severity and complicate clinical diagnosis, particularly in newly affected regions.

## MATERIALS AND METHODS

### Field sites and sampling

Fieldwork was conducted from May to July in each of five consecutive years (2020–2024) at three sites in Maryland, USA: Montgomery County (~39.28°N, −77.10°W), Harford County (~39.58°N, −76.52°W), and Baltimore County (~39.49°N, −76.83°W), as previously described (37). All sites were privately owned properties used for the breeding and housing of hunting hounds, situated within a rural–suburban landscape matrix of farmland, mixed woodland, and maintained grass fields. Each site covered approximately 1.1 ha with a mixture of mature forest, secondary growth, and ecotone habitats with trail systems and fencing throughout. Another site was enrolled in the study in 2022–2024 in Baltimore County. Sites were separated geographically and selected to represent independent field locations within the statewide surveillance network.

Sampling alternated weekly from May to July between small mammal trapping and tick dragging. At each site, linear transects (>5 per site) were established for both trapping and dragging to ensure spatial coverage. For small mammals, 20–40 Sherman live traps (3 × 3 × 10″, Tallahassee, FL, USA) were deployed per site per night and spaced at approximately 15 m intervals. Traps were baited with non-nutritious rodent chow (LabDiet) to lure *P. leucopus*. Traps were set in the evening and checked each morning, non-target species were released immediately, and captured *Peromyscus leucopus* were briefly restrained by scruff for fed-tick removal. Animals were released at the capture site without sedation or euthanasia.

Questing *Ixodes scapularis* nymphs were collected in peak nymphal questing season in Maryland (from mid-May through July) by dragging a 1 m² white flannel cloth across vegetation along woodland and edge transects. Drags were conducted during daylight hours and paused during periods of high heat or low humidity. The cloth was examined at approximately 15 m intervals, and ticks were removed with forceps, placed in labeled tubes, and stored at −20°C. Sampling continued until a minimum threshold of nymphs (*n* = 30) was obtained per site per year to support prevalence estimation. Because drag distance/time and transect-level effort were not recorded in a way that supports density estimation, we do not infer nymph density/burden from these collections.

### Tick identification

All ticks were identified morphologically as *Ixodes scapularis* nymphs. Species identity was confirmed molecularly by PCR amplification of a 152 bp fragment of the actin gene using primers IxAct-F (5′-GCCCTGGACTCCGAGCAG-3′) and IxAct-R (5′-CCGTCGGGAAGCTCGTAGG-3′). Only confirmed nymphs were included in the analyses; no larvae or adults were used.

### DNA extraction

Ticks were surface sterilized sequentially in 10% bleach, 70% ethanol, and sterile PBS, then homogenized individually in 0.5 mL bead-beating tubes prefilled with baked zirconia beads (Glen Mills, Cat. 7361-002000) using a Mini-Beadbeater-24 for 1.5 min. DNA was extracted with a modified Qiagen DNeasy Blood & Tissue Kit (Qiagen, Cat. 1017647) including Proteinase K and carrier RNA. Ear punch and blood samples were treated with extraction buffer, and the lysates were clarified by centrifugation (6,000 × *g*, 10 min), mixed with AL buffer and ethanol, and passed through spin columns with standard wash steps (AW1, AW2, ethanol). DNA was eluted twice in 50 µL AE buffer and stored at −20°C. Negative extraction controls were included once per batch.

### Pathogen detection

Pathogen detection was performed using TaqMan real-time PCR assays. *Borrelia burgdorferi* was detected by a *flaB* TaqMan qPCR (forward primer 5′-AAGCAATCTAGGTCTCAAGC-3′; reverse primer 5′-GCTTCAGCCTGGCCATAAATAG-3′; probe 5′-FAM-AGATGTGGTAGACCCGAAGCCGAG-TAMRA-3′) in 20 µL reactions run on a QuantStudio 7 (Applied Biosystems) with 95°C for 10 min followed by 45 cycles of 95°C for 15 s and 60°C for 1 min. Samples were considered positive if amplification was equivalent to ≥50 *flaB* copies per sample, based on standard curves generated from cultured *B. burgdorferi* DNA.

*Babesia microti* and *Anaplasma phagocytophilum* were detected using a TaqMan multiplex real-time PCR assay (Thermo Scientific reagents) targeting the *B. microti* 18S rRNA and *A. phagocytophilum* 16S rRNA genes, respectively. *B. microti* 18S primers were 5′-GCATGGAATAATGAAGTAGGACTTTGGT-3′ (forward) and 5′-CCCCAACTGCTCCTATTAACCATT-3′ (reverse) with probe 5′-[FAM]-CTCTGGCTCAATAACC-[MGB]-3′. *A. phagocytophilum* 16S primers were 5′-CGGAATTCCTAGTGTAGAGGTGAAA-3′ (forward) and 5′-GTCAGTACCGGACCAGATAGC-3′ (reverse) with probe 5′-[VIC]-CCACTGGTGTTCCTCC-[MGB]-3′. Reactions were run in 20 µL containing 2 µL total DNA extract, 900 nM of each primer, and 250 nM probe, with cycling conditions of 95°C for 20 s followed by 40 cycles of 95°C for 1 s and 60°C for 20 s. The multiplex assay was optimized by comparing singleplex versus multiplex Ct values under identical conditions; once comparable, all samples were run in multiplex. All samples were tested in duplicate, and a Ct ≤ 35 was considered positive. Melt-curve analysis was not performed.

Assay controls included synthetic gBlocks (Integrated DNA Technologies) spanning the complete target regions for *B. burgdorferi flaB*, *B. microti* 18S rRNA, and *A. phagocytophilum* 16S rRNA, together with field-confirmed positive samples. To independently confirm detection and validate the primary qPCR results, 30 qPCR-positive samples per pathogen were re-tested using confirmatory qPCR assays targeting rrf–rrl (5S–23S) intergenic spacer for *Borrelia burgdorferi*, groEL (hsp60) for *Anaplasma phagocytophilum*, and β-tubulin for *Babesia microti*.

### Data handling and statistical analysis

qPCR results were scored positive when amplification occurred within predefined calling thresholds. Coinfection was defined as the simultaneous detection of ≥2 pathogens within the same tick. All analyses were performed in Python v3.12.7 using statsmodels v0.14.2. Infection prevalence was calculated as the proportion positive among tested individuals, stratified by site, year, and sample type (questing nymphs, *P. leucopus*-fed nymphs, and *P. leucopus* hosts), and uncertainty was summarized using 95% Wilson score confidence intervals. Differences in prevalence between groups (e.g., site, year, and collection method) were evaluated using contingency-table tests: Fisher’s exact tests were used when expected cell counts were small; otherwise Pearson’s chi-square tests were used.

Temporal trends were assessed using logistic regression with infection status as the binary outcome and year modeled as a continuous predictor. Models were fit for (i) questing nymphs only (adjusting for site where applicable), (ii) *P. leucopus*-fed nymphs only (site-stratified and pooled, as specified in the Results), and (iii) combined tick data sets including both questing and *P. leucopus*-fed nymphs with covariates for site and collection method. Effect sizes are reported as odds ratios per year with 95% confidence intervals and Wald *P* values. To assess monotonic changes over time in pooled data sets, chi-square tests for linear trend were additionally performed as described in the Results. Coinfection enrichment was evaluated by comparing observed co-detection counts to expected counts under independence within each tick collection method; significance was assessed using Fisher’s exact tests. When multiple hypotheses were tested within a family of comparisons, *P* values were adjusted using Bonferroni and Benjamini–Hochberg false discovery rate procedures, as indicated.

### Human case data

County-level Lyme disease case counts (2014–2023) were obtained from CDC surveillance reporting (county of residence; confirmed/probable per national case definition). County-level anaplasmosis and babesiosis case counts for 2014–2023 were obtained from the Maryland Department of Health (MDH) (38). Data were aggregated by county and year. Population denominators were taken from the U.S. Census Bureau annual estimates to calculate incidence rates. Case definitions followed MDH confirmed/probable classifications. All data used were publicly reportable and contained no personally identifiable information.

## RESULTS

### Questing nymphal infection prevalence

**Fig 1:**
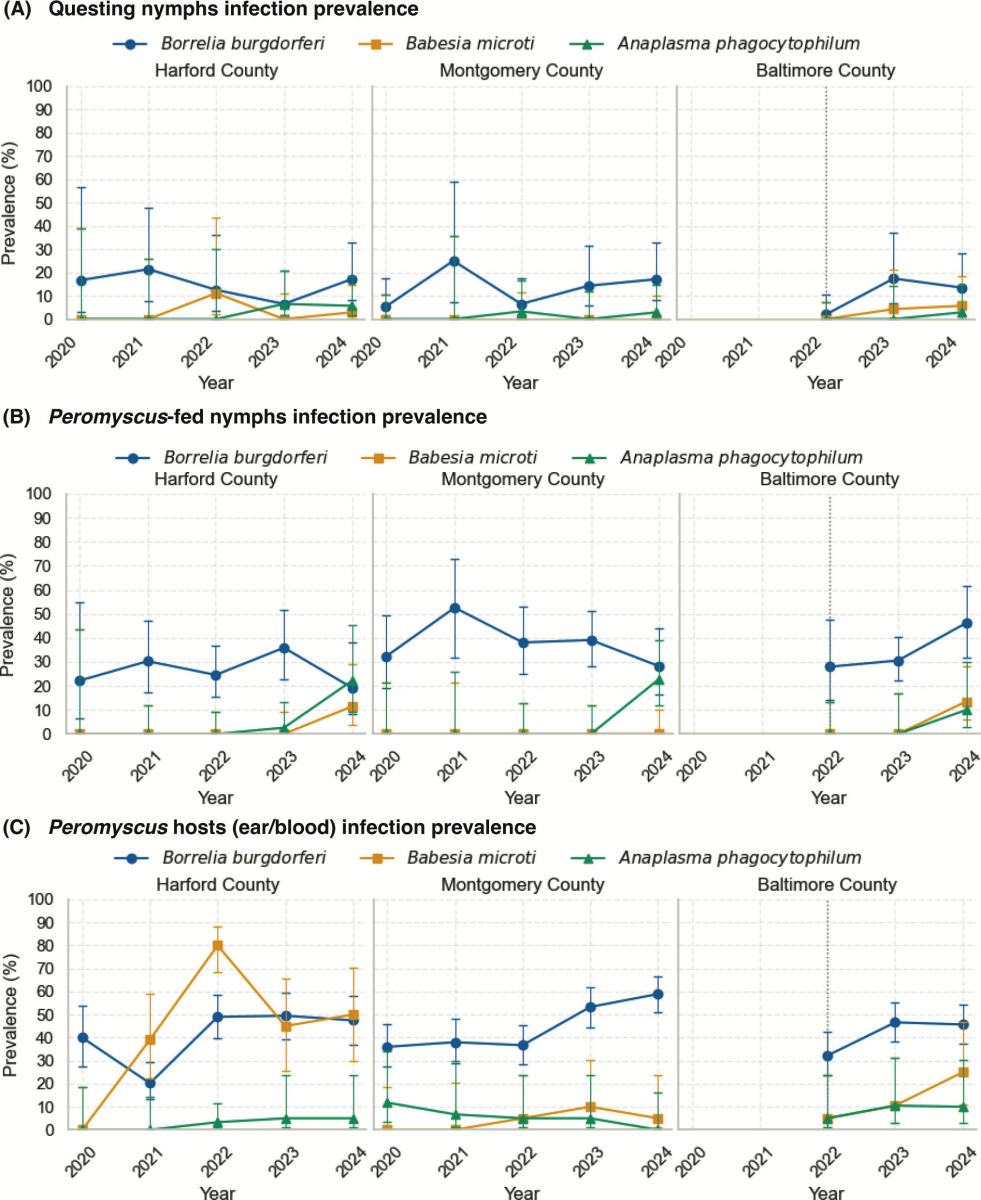
Prevalence of *Borrelia burgdorferi*, *Babesia microti*, and *Anaplasma phagocytophilum* across three field sites in Maryland (2020–2024). (**A**) Flat questing nymphal *Ixodes scapularis*. (**B**) Engorged nymphs collected from trapped *Peromyscus leucopus*. (**C**) *Peromyscus leucopus* hosts. Each site is shown in a separate panel. Prevalence (%) was calculated as the proportion of individuals testing positive for each pathogen by qPCR. For panel C, *B. burgdorferi* prevalence was determined by ear tissue qPCR, while *B. microti* and *A. phagocytophilum* prevalence were determined by blood qPCR. Error bars represent 95% confidence intervals (Wilson’s method).

We evaluated infection prevalence of *Borrelia burgdorferi*, *Babesia microti*, and *Anaplasma phagocytophilum* in questing *Ixodes scapularis* nymphs collected by drag sampling across three Maryland field sites from 2020 to 2024 (Fig. 1A). Across the study, questing-nymph *B. burgdorferi* prevalence ranged from 6.5% to 21.4% in Harford County (*n* = 102), from 5.3% to 25.0% in Montgomery County (*n* = 155), and from 2.0% to 17.4% in Baltimore County (*n* = 111; enrolled 2022–2024) (Fig. 1A). Year-to-year fluctuations were observed within each county, with the higher Montgomery County estimate in 2021 reflecting a smaller sample size (25.0% [2/8]). This relatively stable background is consistent with long-term enzootic maintenance in Maryland.

By contrast, *B. microti* and *A. phagocytophilum* were detected only sporadically and at low prevalence in drag-collected nymphs. In Harford County, *B. microti* was detected in 2022 (11.1% [1/9]) and 2024 (2.9% [1/35]), while *A. phagocytophilum* was detected in 2023 (6.5% [2/31]) and 2024 (5.7% [2/35]). In Montgomery County, *A. phagocytophilum* was detected in 2022 (3.3% [1/30]) and 2024 (2.9% [1/35]), while *B. microti* was not detected in any year tested. In Baltimore County, *B. microti* was detected in 2023 (4.3% [1/23]) and 2024 (5.7% [2/35]), whereas *A. phagocytophilum* was detected once in 2024 (2.9% [1/35]). Logistic regression adjusting for site suggested a modest, non-significant increase over time for *B. microti* (OR per year = 1.96, 95% CI: 0.67–5.76, *P* = 0.22) and a stronger but still non-significant upward signal for *A. phagocytophilum* (OR per year = 2.15, 95% CI: 0.89–5.22, *P* = 0.089). Pooled chi-square tests for trend did not detect significant monotonic increases (*B. microti*, *P* = 0.60; *A. phagocytophilum*, *P* = 0.41). Together, these results highlight sporadic detections with county-specific and year-specific detections rather than smooth linear increases as observed for *B. burgdorferi*.

### *P. leucopus*-fed nymph infection prevalence

Infection prevalence in *P. leucopus*-fed nymphs (Fig. 1B) was consistently higher than in questing ticks, particularly for *B. burgdorferi*. Across sites and years, *B. burgdorferi* prevalence typically ranged from ~19% to 53% in *P. leucopus*-fed nymphs, about 2–3× higher than in drag-collected nymphs (compare with Fig. 1A), reflecting additional acquisition of *B. burgdorferi* from infected mice. Because these nymphs contain a recent blood meal, detections—particularly for *A. phagocytophilum*—may in some cases reflect blood meal-associated DNA rather than fully established infection in tick tissues. *B. burgdorferi* screening was performed on all *P. leucopus*-fed nymphs collected (Harford County, *n* = 168; Montgomery County, *n* = 198; and Baltimore County, *n* = 162), and county-specific ranges were 19.2%–35.9% in Harford County (19.2% [5/26] in 2024 to 35.9% [14/39] in 2023), 28.2%–52.6% in Montgomery County (28.2% [11/39] in 2024 to 52.6% [10/19] in 2021), and 28.0%–46.2% in Baltimore County (enrolled 2022–2024; 28.0% [7/25] in 2022 to 46.2% [18/39] in 2024).

Testing for *B. microti* and *A. phagocytophilum* was performed on a subset of *P. leucopus*-fed nymphs (multiplex-tested denominators are shown), and detections were generally later and more sporadic than for *B. burgdorferi. B. microti* was detected in 2024 in Harford County (11.5% [3/26 tested]) and Baltimore County (13.5% [5/37 tested]) and was not detected in Montgomery County in any year tested. *A. phagocytophilum* was uncommon early (e.g., Harford County 2023: 2.6% [1/38 tested]) but reached higher prevalence in 2024 in Harford County (22.2% [4/18 tested]) and Montgomery County (22.9% [8/35 tested]), with lower but nonzero detections in Baltimore County (2024: 10.0% [2/20 tested]). Notably, *A. phagocytophilum* prevalence in *P. leucopus*-fed nymphs was nearly an order of magnitude higher than in questing ticks, exceeding 20% in some county–years (Fig. 1B). At the Harford County site, logistic regression revealed a statistically significant annual increase (OR = 8.81, 95% CI: 1.23–63.03, *P* = 0.0302). A combined statewide model across all sites showed an even stronger effect (OR = 16.18, 95% CI: 2.37–110.67, *P* = 0.0045), supporting active expansion of *A. phagocytophilum* within rodent-associated tick populations. These patterns indicate that *A. phagocytophilum* is circulating robustly in rodent hosts and is detected readily in feeding nymphs before it becomes apparent in the questing population, consistent with an early, patchy stage of establishment.

### Combined trend analysis across ticks

When data from both questing and *P. leucopus-*fed nymphs were analyzed together, strong evidence emerged for increasing prevalence of *B. microti* and *A. phagocytophilum* over time. Logistic regression adjusting for site and collection method indicated significant year-on-year increases: *B. microti* prevalence rose approximately 4.7-fold per year (OR = 4.7, 95% CI: 1.6–13.7, *P* = 0.004), while *A. phagocytophilum* rose approximately 5.4-fold per year (OR = 5.4, 95% CI: 2.3–12.5, *P* < 0.001).

Pooled chi-square trend tests confirmed these increases (*B. microti*, *P* = 0.0009; *A. phagocytophilum*, *P* = 6.8 × 10⁻⁶). Site effects were consistent with the individual analyses: Harford and Montgomery Counties generally showed higher *A. phagocytophilum* prevalence relative to Baltimore County. Collection method (questing versus *P. leucopus*-fed) was not significant for *B. microti* but approached significance for *A. phagocytophilum*, consistent with stronger inference from *P. leucopus*-fed ticks.

### *Peromyscus leucopus* infection prevalence

Infections in *P. leucopus* provided the strongest evidence of enzootic circulation, with host prevalences generally exceeding those observed in ticks (Fig. 1C). *B. burgdorferi* (ear tissue qPCR) was consistently detected across counties and years, with county-specific prevalence ranges of 39.4%–50.0% in Harford County (annual *n* = 50–102), 32.9%–58.9% in Montgomery County (annual *n* = 92–146), and 32.2%–46.6% in Baltimore County (annual *n* = 90–133; enrolled 2022–2024), consistent with long-standing enzootic maintenance (Fig. 1C).

For *B. microti* and *A. phagocytophilum* (blood qPCR; blood denominators apply), host infection was more heterogeneous. *B. microti* showed pronounced site-specific and year-specific peaks, most notably in Harford County (80.0% [12/15] in 2022; 50.0% [8/16] in 2024), with lower prevalence in Montgomery County (0%–9.1% across years; e.g., 9.1% [1/11] in 2022) and a gradual increase in Baltimore County (5.0% [1/20] in 2022 to 25.0% [5/20] in 2024). *A. phagocytophilum* was generally detected at low-to-moderate prevalence, peaking in Harford County (22.2% [4/18] in 2024) and remaining lower in Montgomery County (0%–11.8% across years; including 0% [0/17] in 2024) and Baltimore County (5.0%–10.5% across years; e.g., 10.5% [2/19] in 2023). Importantly, host detections of *B. microti* and *A. phagocytophilum* occurred in multiple county–years where questing-nymph prevalence was 0% (0/*x* tested), supporting rodent surveillance as a leading indicator of early enzootic establishment.

### Coinfection analysis

Coinfections among *Ixodes scapularis* nymphs were rare (Table 1). Most ticks carried a single pathogen, and coinfection combinations occurred only sporadically.

**TABLE 1:** Observed and expected coinfections in *Ixodes scapularis* nymphs by collection method^*a*^

Coinfection type	Method	*n*	Observed count	Observed % of ticks	Expected count	Fisher’s exact *P*
*B. burgdorferi + A. phagocytophilum*	Drag	333	2	0.60	0.82	0.192
*B. burgdorferi + A. phagocytophilum*	*P. leucopus*-fed	332	9	2.71	5.02	0.023
*B. burgdorferi + B. microti*	Drag	333	0	0.00	0.59	1
*B. burgdorferi + B. microti*	*P. leucopus*-fed	332	5	1.51	2.67	0.123
*A. phagocytophilum + B. microti*	Drag	333	0	0.00	0.11	1
*A. phagocytophilum + B. microti*	*P. leucopus*-fed	332	1	0.30	0.36	0.312
Triple (*B.b. + A.p. + B*.*m*.)	Drag	333	0	0.00	0.01	N/A^*b*^
Triple (*B.b. + A.p. + B*.*m*.)	*P. leucopus*-fed	332	1	0.30	0.12	N/A

^*a*^ Observed and expected coinfection frequencies of *Borrelia burgdorferi*, *Anaplasma phagocytophilum*, and *Babesia microti* in drag-collected nymphs versus *Peromyscus leucopus*-fed nymphs across three Maryland field sites (2020–2024). Expected counts were calculated under independence; *P* values are from Fisher’s exact tests.

^*b*^ N/A indicates that no *P* value was calculated for the triple-infection category; only one triple-infected tick was observed overall (0/333 drag-collected nymphs and 1/332 *P. leucopus*-fed nymphs), making statistical comparison not meaningful.

The only statistically significant enrichment was *B. burgdorferi + A. phagocytophilum* coinfections in *P. leucopus-*fed nymphs, detected in 9 ticks (2.71%) compared to an expected 5.0 under independence (*P* = 0.023). This enrichment may reflect ecological facilitation via shared hosts or overlapping seasonal transmission windows. Other coinfection types were either absent or occurred at chance levels. *B. burgdorferi + B. microti* coinfections were detected five times in *P. leucopus-*fed ticks and not at all in questing ticks. *A. phagocytophilum + B. microti* coinfections were observed once in *P. leucopus*-fed and not in questing ticks. A single triple coinfection (*B. burgdorferi + A. phagocytophilum + B. microti*) was detected in a single *P. leucopus-*fed nymph but was too rare for statistical analysis.

### Human case trends of emerging tick-borne diseases

**Fig 2:**
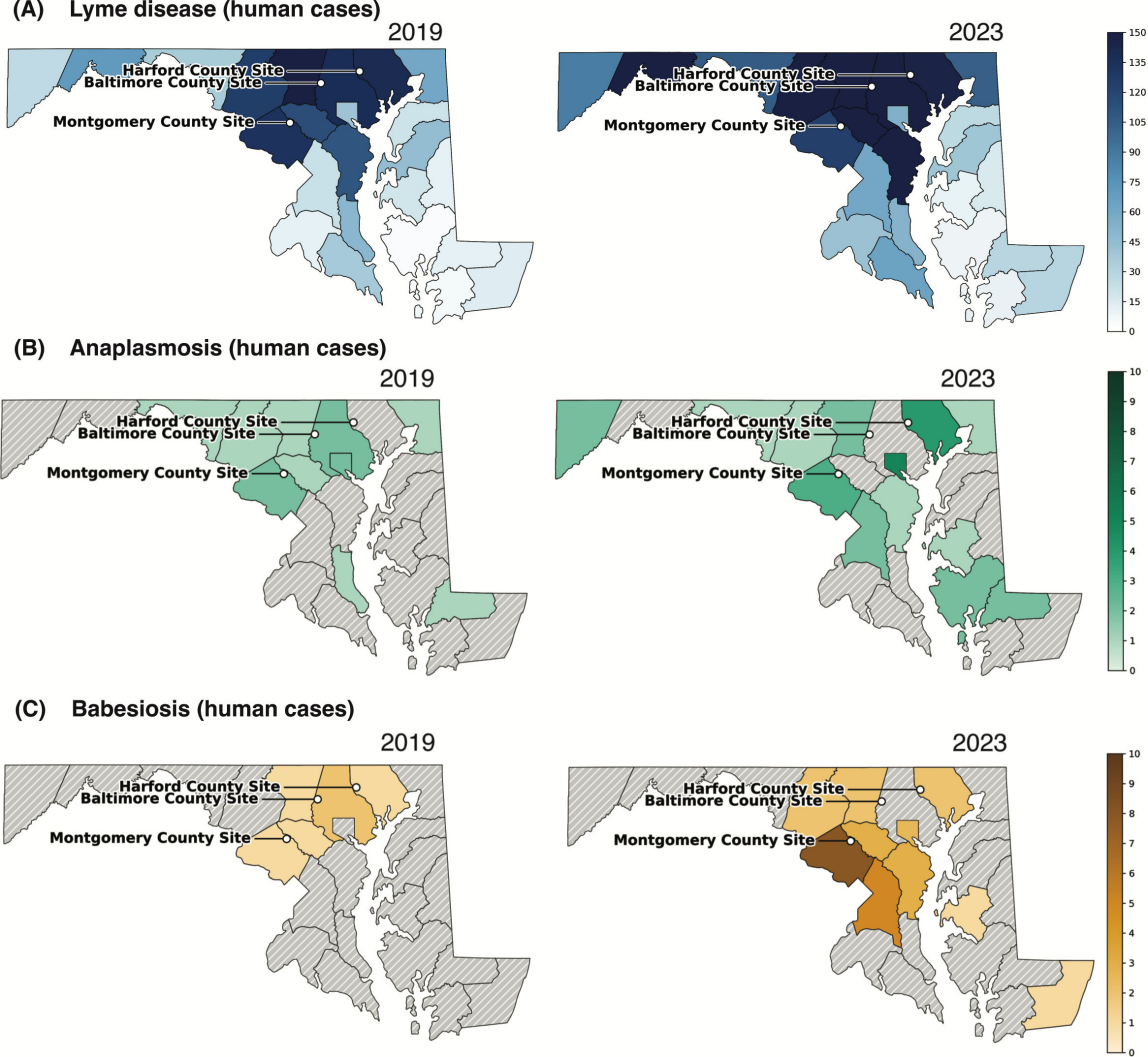
Human tick-borne disease cases in Maryland. Shown are annual confirmed human cases by county for two representative years from the study period: 2019 (left) and 2023 (right). (**A**) Lyme disease cases. (**B**) Anaplasmosis cases. (**C**) Babesiosis cases. Note that the color scale in panel A differs from that in panels B and C to reflect the higher case burden for Lyme disease. Case data were obtained from CDC (Lyme disease) and Maryland Department of Health (human anaplasmosis and babesiosis) surveillance records. Maps were generated in Python (GeoPandas/Matplotlib).

County-level confirmed human case counts are shown for two representative years from the study period (2019 and 2023; Fig. 2A through C; Table S2; Maryland Department of Health [38]). These surveillance data are reported at a broader spatial scale than our site-level collections, and annual case counts for anaplasmosis and babesiosis remain low in many county–year strata; together with sampling at only three field sites, this limits power and makes formal statistical linkage inappropriate. We therefore present county-level patterns for qualitative context alongside enzootic detections in ticks and *Peromyscus* hosts.

Lyme disease case counts were substantially higher and more widespread than anaplasmosis or babesiosis in both 2019 and 2023 (Fig. 2A), consistent with long-standing statewide endemicity and providing context for interpreting the comparatively low but increasing burden of the emerging pathogens.

By contrast, anaplasmosis and babesiosis remained comparatively rare but showed a clearer emergence signal after 2019 (Fig. 2B and C). For anaplasmosis, statewide totals rose to 29 cases in 2022–2023, with repeated reports in Montgomery County (2 cases in 2019; 3 in 2023) and increasing reports in Harford County (0 in 2019; 4 in 2023). Baltimore City reported 5 cases in 2019 and 4 in 2023, whereas Baltimore County reported 2 cases in 2019 and none from 2020 to 2023.

Babesiosis showed a similar but slightly delayed pattern. Statewide totals were ≤3 per year during 2014–2018 but increased to 27 cases by 2023. Montgomery County again showed the strongest signal (1 case in 2019; 8 in 2023), Harford County reported cases in both 2019 and 2023 (1 and 2, respectively), and Baltimore County illustrated continued patchiness (2 cases in 2019; none in 2023 despite intermittent low-level reports in earlier years).

Within these constraints, the timing and geography of county-level reports are directionally consistent with enzootic patterns observed in *Peromyscus* hosts and *P. leucopus*-fed nymphs: Montgomery and Harford Counties show repeated human reports during the period when we more frequently detect *A. phagocytophilum* and *B. microti* in rodents and host-fed ticks, whereas Baltimore County underscores spatial heterogeneity. Together, these observations support strengthening local transmission in parts of Maryland while emphasizing that both enzootic detections and human case reporting remain heterogeneous across space and time.

## DISCUSSION

### Emergence in a *Borrelia-*endemic landscape

Predicting pathogen emergence in transitional zones requires surveillance methods that detect early signals before pathogens become established in questing tick populations. In Maryland, *Borrelia burgdorferi* is long established (39–41), but our 5-year surveillance reveals increasing yet incomplete establishment of *Babesia microti* and *Anaplasma phagocytophilum*. The strongest signals appear in *Peromyscus leucopus* hosts and *P. leucopus-*fed nymphs, indicating active but still developing enzootic cycles.

### Biological constraints and contrasting establishment

*B. burgdorferi* maintains enzootic cycles due to its efficient persistence in the tick midgut and regulated migration to salivary glands during feeding (42–44). In contrast, *A. phagocytophilum* must colonize midgut cells and hemocytes and manipulate tick immune processes (45–51), creating multiple bottlenecks that may explain low prevalence in questing ticks even when host infection is present. *B. microti* must undergo sexual development in the tick after acquisition and lacks transovarial transmission (11, 12, 29). This dependency on reacquisition in each generation, combined with reduced metabolic flexibility (52, 53), yields inefficient transmission and contributes to patchier establishment. These life-cycle differences help explain why *B. microti* and *A. phagocytophilum* initially emerge more strongly in hosts and feeding ticks than in questing ticks. In addition, vertical (transplacental) transmission in reservoir hosts has been described for *B. microti* and may contribute to elevated host prevalence without proportional amplification in tick populations, further decoupling host and vector infection dynamics, as observed here, where host prevalence markedly exceeded that of questing ticks (54).

### *P. leucopus-*fed nymphs as early-warning xenodiagnostic indicators

In multiple site-years, *A. phagocytophilum* prevalence in *P. leucopus-*fed nymphs exceeded 20%, nearly order-of-magnitude higher than in questing ticks. Some detections likely reflect DNA in the blood meal rather than stable tick tissue infection (55). Mammalian permissiveness, including immune dampening in dogs and mice (6, 27), may support high host infection without efficient vector persistence. Regardless of the mechanism, these findings demonstrate that *P. leucopus-*fed ticks capture early pathogen circulation that would be underestimated using drag sampling alone.

Rising Maryland human cases from 2019 to 2023, especially in Montgomery County, reinforce that *P. leucopus-*fed tick surveillance can serve as a xenodiagnostic indicator of establishment.

### Coinfection dynamics during early establishment

Coinfections were rare overall, consistent with incomplete emergence of *B. microti* and *A. phagocytophilum*. The only statistically significant association was enrichment of *B. burgdorferi + A. phagocytophilum* coinfections in *P. leucopus-*fed nymphs. Conversely, *B. burgdorferi + B. microti* coinfections, widely documented in ecological (14, 49, 56, 57) and clinical studies (26, 58), were scarce. One hypothesis is that early establishment of *A. phagocytophilum* overlaps more directly with established enzootic *B. burgdorferi* cycles in *P. leucopus*, whereas *B. microti* arrival is more recent and stochastic. The absence of *A. phagocytophilum + B. microti* combinations may reflect suppressive dynamics observed in experimental models (15, 29, 59).

### Public health implications

Although absolute Maryland case numbers remain low, both anaplasmosis and babesiosis show clear upward trends. *B. microti* is the leading transfusion-transmitted parasite in the United States (60–62), and *A. phagocytophilum* can cause severe disease in high-risk populations (7, 27, 63). The concordance of rodent, tick-fed, and human case trends suggests strengthening local transmission and highlights the need for proactive surveillance. Although surveillance scales differ and county case counts are low and patchy for anaplasmosis and babesiosis, the timing of repeated county-level reports in Montgomery and Harford Counties is directionally consistent with increased detections in *P. leucopus* and *P. leucopus*-fed nymphs, supporting strengthening local transmission in parts of Maryland while emphasizing spatial heterogeneity (e.g., Baltimore County).

### Regional context and ecological drivers

Emergence patterns in Maryland mirror historical trajectories in the Northeast and Upper Midwest, where *B. microti* and *A. phagocytophilum* lagged decades behind *B. burgdorferi* establishment (12, 18, 21, 64, 65). Environmental factors, including land-use change, deer abundance, mast cycles, and climate warming, shape tick and pathogen dynamics (66–69). As a transitional ecotone adjoining long-standing endemic foci in the Northeast, Maryland appears poised for continued pathogen spread southward and westward.

### Limitations and future directions

This study did not characterize pathogen genotypes nor confirm tissue-established tick infection. Priority next steps include integrating strain-level analyses, confirmation of salivary gland colonization particularly for *A. phagocytophilum* ecotypes (36), and expansion across the broader mid-Atlantic to assess geographic generalizability.

### Conclusion

*B. burgdorferi* remains deeply established in Maryland, whereas *B. microti* and *A. phagocytophilum* are clearly emerging. *P. leucopus-*fed ticks provide an early-warning signal that precedes widespread establishment in questing ticks and should be incorporated into routine surveillance as a xenodiagnostic tool. Integrated monitoring across hosts, fed ticks, and questing ticks will be essential for anticipating tick-borne disease emergence in transitional landscapes.

## SUPPLEMENTAL MATERIAL

Supplemental material aem.02242-25-s0001.docx Tables S1 to S3; Fig. S1.

ASM does not own the copyrights to Supplemental Material that may be linked to, or accessed through, an article. The authors have granted ASM a non-exclusive, world-wide license to publish the Supplemental Material files. Please contact the corresponding author directly for reuse.

## References

[1] Sonenshine DE. 2018. Range expansion of tick disease vectors in North America: implications for spread of tick-borne disease. Int J Environ Res Public Health 15:478. doi:10.3390/ijerph15030478 29522469 PMC5877023

[2] Eisen RJ, Paddock CD. 2021. Tick and tickborne pathogen surveillance as a public health tool in the United States. J Med Entomol 58:1490–1502. doi:10.1093/jme/tjaa087 32440679 PMC8905548

[3] Hersh MH, Ostfeld RS, McHenry DJ, Tibbetts M, Brunner JL, Killilea ME, LoGiudice K, Schmidt KA, Keesing F. 2014. Co-infection of blacklegged ticks with Babesia microti and Borrelia burgdorferi is higher than expected and acquired from small mammal hosts. PLoS One 9:e99348. doi:10.1371/journal.pone.0099348 24940999 PMC4062422

[4] Randolph SE. 2001. The shifting landscape of tick-borne zoonoses: tick-borne encephalitis and Lyme borreliosis in Europe. Philos Trans R Soc Lond B Biol Sci 356:1045–1056. doi:10.1098/rstb.2001.0893 11516382 PMC1088499

[5] Goethert HK, Molloy P, Berardi V, Weeks K, Telford SR. 2018. Zoonotic Babesia microti in the northeastern U.S.: Evidence for the expansion of a specific parasite lineage. PLoS One 13:e0193837. doi:10.1371/journal.pone.0193837 29565993 PMC5864094

[6] Vannier E, Krause PJ. 2012. Human babesiosis. N Engl J Med 366:2397–2407. doi:10.1056/NEJMra1202018 22716978

[7] Bakken JS, Dumler JS. 2015. Human granulocytic anaplasmosis. Infect Dis Clin North Am 29:341–355. doi:10.1016/j.idc.2015.02.007 25999228 PMC4441757

[8] Barbour AG, Fish D. 1993. The biological and social phenomenon of Lyme disease. Science 260:1610–1616. doi:10.1126/science.8503006 8503006

[9] Eisen L. 2023. Rodent-targeted approaches to reduce acarological risk of human exposure to pathogen-infected Ixodes ticks. Ticks Tick Borne Dis 14:102119. doi:10.1016/j.ttbdis.2023.102119 36680999 PMC10863499

[10] Tufts DM, Goethert HK, Diuk-Wasser MA. 2024. Host-pathogen associations inferred from bloodmeal analyses of Ixodes scapularis ticks in a low biodiversity setting. Appl Environ Microbiol 90:e0066724. doi:10.1128/aem.00667-24 39207157 PMC11409645

[11] Mather TN, Telford SR III, Moore SI, Spielman A. 1990. Borrelia burgdorferi and Babesia microti: efficiency of transmission from reservoirs to vector ticks (Ixodes dammini). Exp Parasitol 70:55–61. doi:10.1016/0014-4894(90)90085-Q 2295326

[12] Spielman A, Wilson ML, Levine JF, Piesman J. 1985. Ecology of Ixodes dammini-borne human babesiosis and Lyme disease. Annu Rev Entomol 30:439–460. doi:10.1146/annurev.en.30.010185.002255 3882050

[13] Dunn JM, Krause PJ, Davis S, Vannier EG, Fitzpatrick MC, Rollend L, Belperron AA, States SL, Stacey A, Bockenstedt LK, Fish D, Diuk-Wasser MA. 2015. Borrelia burgdorferi promotes the establishment of Babesia microti in the northeastern United States. PLoS One 9:e115494. doi:10.1371/journal.pone.0115494 PMC4278703 25545393

[14] Diuk-Wasser MA, Vannier E, Krause PJ. 2016. Coinfection by Ixodes tick-borne pathogens: ecological, epidemiological, and clinical consequences. Trends Parasitol 32:30–42. doi:10.1016/j.pt.2015.09.008 26613664 PMC4713283

[15] Rocha SC, Moustafa MAM, Velásquez CV, Azuama OC, Zafar K, Meyer C, Araujo M, Taylor K, Parveen N. 2025. Long-term survival of Babesia microti and Borrelia burgdorferi in C3H/HeJ mice and their effect on Lyme arthritis and babesiosis manifestations. Microbiol Spectr 13:e0025225. doi:10.1128/spectrum.00252-25 40793757 PMC12403850

[16] Foley J, Nieto NC, Madigan J, Sykes J. 2008. Possible differential host tropism in Anaplasma phagocytophilum strains in the Western United States. Ann N Y Acad Sci 1149:94–97. doi:10.1196/annals.1428.066 19120182

[17] Kocan KM, Blouin EF, Barbet AF. 2000. Anaplasmosis control. Past, present, and future. Ann N Y Acad Sci 916:501–509. doi:10.1111/j.1749-6632.2000.tb05329.x 11193665

[18] Holman MS, Caporale DA, Goldberg J, Lacombe E, Lubelczyk C, Rand PW, Smith RP. 2004. Anaplasma phagocytophilum, Babesia microti, and Borrelia burgdorferi in Ixodes scapularis, southern coastal Maine. Emerg Infect Dis 10:744–746. doi:10.3201/eid1004.030566 15200875 PMC3323092

[19] Hutchinson ML, Strohecker MD, Simmons TW, Kyle AD, Helwig MW. 2015. Prevalence rates of Borrelia burgdorferi (Spirochaetales: Spirochaetaceae), Anaplasma phagocytophilum (Rickettsiales: Anaplasmataceae), and Babesia microti (Piroplasmida: Babesiidae) in host-seeking Ixodes scapularis (Acari: Ixodidae) from Pennsylvania. J Med Entomol 52:693–698. doi:10.1093/jme/tjv037 26335476

[20] Hodzic E, Fish D, Maretzki CM, De Silva AM, Feng S, Barthold SW. 1998. Acquisition and transmission of the agent of human granulocytic ehrlichiosis by Ixodes scapularis ticks. J Clin Microbiol 36:3574–3578. doi:10.1128/JCM.36.12.3574-3578.1998 9817875 PMC105242

[21] Massung RF, Levin ML, Munderloh UG, Silverman DJ, Lynch MJ, Gaywee JK, Kurtti TJ. 2007. Isolation and propagation of the Ap-Variant 1 strain of Anaplasma phagocytophilum in a tick cell line. J Clin Microbiol 45:2138–2143. doi:10.1128/JCM.00478-07 17475757 PMC1932999

[22] Knapp KL, Rice NA. 2015. Human coinfection with Borrelia burgdorferi and Babesia microti in the United States. J Parasitol Res 2015:587131. doi:10.1155/2015/587131 26697208 PMC4677215

[23] Hoversten K, Bartlett MA. 2018. Diagnosis of a tick-borne coinfection in a patient with persistent symptoms following treatment for Lyme disease. BMJ Case Rep 2018:bcr2018225342. doi:10.1136/bcr-2018-225342 PMC6169620 30262525

[24] Saetre K, Godhwani N, Maria M, Patel D, Wang G, Li KI, Wormser GP, Nolan SM. 2018. Congenital babesiosis after maternal infection with Borrelia burgdorferi and Babesia microti. J Pediatric Infect Dis Soc 7:e1–e5. doi:10.1093/jpids/pix074 28992325

[25] Martínez-Balzano C, Hess M, Malhotra A, Lenox R. 2015. Severe babesiosis and Borrelia burgdorferi co-infection. QJM 108:141–143. doi:10.1093/qjmed/hcs100 22685248

[26] Krause PJ, Telford SR 3rd, Spielman A, Sikand V, Ryan R, Christianson D, Burke G, Brassard P, Pollack R, Peck J, Persing DH. 1996. Concurrent Lyme disease and babesiosis. Evidence for increased severity and duration of illness. JAMA 275:1657–1660. doi:10.1001/jama.1996.03530450047031 8637139

[27] Moniuszko-Malinowska A, Dunaj J, Andersson MO, Chmielewski T, Czupryna P, Groth M, Grygorczuk S, Zajkowska J, Kondrusik M, Kruszewska E, Pancewicz S. 2021. Anaplasmosis in Poland - analysis of 120 patients. Ticks Tick Borne Dis 12:101763. doi:10.1016/j.ttbdis.2021.101763 34161867

[28] Schulze TL, Jordan RA, Healy SP, Roegner VE. 2013. Detection of Babesia microti and Borrelia burgdorferi in host-seeking Ixodes scapularis (Acari: Ixodidae) in Monmouth County, New Jersey. J Med Entomol 50:379–383. doi:10.1603/me12088 23540127

[29] Tufts DM, Adams B, Diuk-Wasser MA. 2023. Ecological interactions driving population dynamics of two tick-borne pathogens, Borrelia burgdorferi and Babesia microti. Proc Biol Sci 290:20230642. doi:10.1098/rspb.2023.0642 37357860 PMC10291726

[30] Walk ST, Xu G, Stull JW, Rich SM. 2009. Correlation between tick density and pathogen endemicity, New Hampshire. Emerg Infect Dis 15:585–587. doi:10.3201/eid1504.080940 19331738 PMC2671416

[31] Anderson JF, Johnson RC, Magnarelli LA, Hyde FW, Myers JE. 1986. Peromyscus leucopus and Microtus pennsylvanicus simultaneously infected with Borrelia burgdorferi and Babesia microti. J Clin Microbiol 23:135–137. doi:10.1128/jcm.23.1.135-137.1986 3517038 PMC268587

[32] Diuk-Wasser MA, Hoen AG, Cislo P, Brinkerhoff R, Hamer SA, Rowland M, Cortinas R, Vourc’h G, Melton F, Hickling GJ, Tsao JI, Bunikis J, Barbour AG, Kitron U, Piesman J, Fish D. 2012. Human risk of infection with Borrelia burgdorferi, the Lyme disease agent, in eastern United States. Am J Trop Med Hyg 86:320–327. doi:10.4269/ajtmh.2012.11-0395 22302869 PMC3269287

[33] Dill GM, Rounsville TF, Bryant AM, Groden E, Gardner AM. 2024. Effects of Peromyscus spp. (Rodentia: Cricetidae) presence, land use, and ecotone on Ixodes scapularis (Acari: Ixodidae) ecology in an emergent area for tick-borne disease. J Med Entomol 61:1478–1488. doi:10.1093/jme/tjae113 39214519 PMC11562968

[34] CDC. 2023. Babesiosis surveillance – United States, 2011–2019. Available from: https://www.cdc.gov/babesiosis/php/datastats/index.html 10.15585/mmwr.ss6806a1 31145719

[35] Stromdahl EY, Feldman KA, Nadolny RM, Kennedy AC, Bement ZJ, Buoni M, et al.. 2009. Emerging babesiosis in the mid-Atlantic: autochthonous human babesiosis cases and Babesia microti (Piroplasmida: Babesiidae) in Ixodes scapularis (Acari: Ixodidae) and Ixodes keiransi (Acari: Ixodidae) ticks from Delaware. J Med Entomol 62:995–1008. doi:10.1093/jme/tjaf054 40261095

[36] Goethert HK, Telford SR. 2022. Host contributions to the force of Borrelia burgdorferi and Babesia microti transmission differ at edges of and within a small habitat patch. Appl Environ Microbiol 88:e0239121. doi:10.1128/aem.02391-21 34985986 PMC8939355

[37] Poje JE, Azevedo JF, Nair N, Mahachi K, Frank LE, Sherpa P, Krizek RS, Baccam T, Gomes-Solecki M, Petersen CA. 2022. Borrelia burgdorferi (Spirochaetales: Spirochaetaceae) infection prevalence and host associations of ticks found on Peromyscus spp. in Maryland. J Med Entomol 59:752–757. doi:10.1093/jme/tjab206 34971369 PMC8924970

[38] Health MDo. 2025. Disease case counts and rates by County Baltimore, MD: Maryland Department of Health. Available from: https://health.maryland.gov/phpa/OIDEOR/CIDSOR/Pages/disease-conditions-countrates.aspx

[39] Amerasinghe FP, Breisch NL, Neidhardt K, Pagac B, Scott TW. 1993. Increasing density and Borrelia burgdorferi infection of deer-infesting Ixodes dammini (Acari: Ixodidae) in Maryland. J Med Entomol 30:858–864. doi:10.1093/jmedent/30.5.858 8254631

[40] Hofmeister EK, Ellis BA, Glass GE, Childs JE. 1999. Longitudinal study of infection with Borrelia burgdorferi in a population of Peromyscus leucopus at a Lyme disease-enzootic site in Maryland. Am J Trop Med Hyg 60:598–609. doi:10.4269/ajtmh.1999.60.598 10348235

[41] Anderson JM, Norris DE. 2006. Genetic diversity of Borrelia burgdorferi sensu stricto in Peromyscus leucopus, the primary reservoir of Lyme disease in a region of endemicity in southern Maryland. Appl Environ Microbiol 72:5331–5341. doi:10.1128/AEM.00014-06 16885284 PMC1538722

[42] Schwan TG, Piesman J. 2000. Temporal changes in outer surface proteins A and C of the lyme disease-associated spirochete, Borrelia burgdorferi, during the chain of infection in ticks and mice. J Clin Microbiol 38:382–388. doi:10.1128/JCM.38.1.382-388.2000 10618120 PMC88728

[43] Grimm D, Tilly K, Byram R, Stewart PE, Krum JG, Bueschel DM, Schwan TG, Policastro PF, Elias AF, Rosa PA. 2004. Outer-surface protein C of the Lyme disease spirochete: a protein induced in ticks for infection of mammals. Proc Natl Acad Sci USA 101:3142–3147. doi:10.1073/pnas.0306845101 14970347 PMC365757

[44] Anguita J, Hedrick MN, Fikrig E. 2003. Adaptation of Borrelia burgdorferi in the tick and the mammalian host. FEMS Microbiol Rev 27:493–504. doi:10.1016/S0168-6445(03)00036-6 14550942

[45] Ramasamy E, Taank V, Anderson JF, Sultana H, Neelakanta G. 2020. Repression of tick microRNA-133 induces organic anion transporting polypeptide expression critical for Anaplasma phagocytophilum survival in the vector and transmission to the vertebrate host. PLoS Genet 16:e1008856. doi:10.1371/journal.pgen.1008856 32614824 PMC7331985

[46] Rolandelli A, Laukaitis-Yousey HJ, Bogale HN, Singh N, Samaddar S, O’Neal AJ, Ferraz CR, Butnaru M, Mameli E, Xia B, Mendes MT, Butler LR, Marnin L, Cabrera Paz FE, Valencia LM, Rana VS, Skerry C, Pal U, Mohr SE, Perrimon N, Serre D, Pedra JHF. 2024. Tick hemocytes have a pleiotropic role in microbial infection and arthropod fitness. Nat Commun 15:2117. doi:10.1038/s41467-024-46494-3 38459063 PMC10923820

[47] Alberdi P, Mansfield KL, Manzano-Román R, Cook C, Ayllón N, Villar M, Johnson N, Fooks AR, de la Fuente J. 2016. Tissue-specific signatures in the transcriptional response to Anaplasma phagocytophilum infection of Ixodes scapularis and Ixodes ricinus tick cell lines. Front Cell Infect Microbiol 6:20. doi:10.3389/fcimb.2016.00020 26904518 PMC4748044

[48] Villar M, Ayllón N, Alberdi P, Moreno A, Moreno M, Tobes R, Mateos-Hernández L, Weisheit S, Bell-Sakyi L, de la Fuente J. 2015. Integrated metabolomics, transcriptomics and proteomics identifies metabolic pathways affected by Anaplasma phagocytophilum infection in tick cells. Mol Cell Proteomics 14:3154–3172. doi:10.1074/mcp.M115.051938 26424601 PMC4762615

[49] Hersh MH, Tibbetts M, Strauss M, Ostfeld RS, Keesing F. 2012. Reservoir competence of wildlife host species for Babesia microti. Emerg Infect Dis 18:1951–1957. doi:10.3201/eid1812.111392 23171673 PMC3557901

[50] Sultana H, Neelakanta G, Kantor FS, Malawista SE, Fish D, Montgomery RR, Fikrig E. 2010. Anaplasma phagocytophilum induces actin phosphorylation to selectively regulate gene transcription in Ixodes scapularis ticks. J Exp Med 207:1727–1743. doi:10.1084/jem.20100276 20660616 PMC2916137

[51] Turck JW, Sultana H, Neelakanta G. 2025. Arthropod autophagy molecules facilitate Anaplasma phagocytophilum infection of Ixodes scapularis tick cells. Commun Biol 8:433. doi:10.1038/s42003-025-07859-6 40082564 PMC11906822

[52] Cornillot E, Hadj-Kaddour K, Dassouli A, Noel B, Ranwez V, Vacherie B, Augagneur Y, Brès V, Duclos A, Randazzo S, et al.. 2012. Sequencing of the smallest Apicomplexan genome from the human pathogen Babesia microti. Nucleic Acids Res 40:9102–9114. doi:10.1093/nar/gks700 22833609 PMC3467087

[53] Puri A, Bajpai S, Meredith S, Aravind L, Krause PJ, Kumar S. 2021. Babesia microti: pathogen genomics, genetic variability, immunodominant antigens, and pathogenesis. Front Microbiol 12:697669. doi:10.3389/fmicb.2021.697669 34539601 PMC8446681

[54] Tufts DM, Diuk-Wasser MA. 2021. Vertical transmission: a vector-independent transmission pathway of Babesia microti in the natural reservoir host Peromyscus leucopus. J Infect Dis 223:1787–1795. doi:10.1093/infdis/jiaa595 32959880 PMC8161636

[55] Răileanu C, Silaghi C, Fingerle V, Margos G, Thiel C, Pfister K, Overzier E. 2021. Borrelia burgdorferi sensu lato in questing and engorged ticks from different habitat types in southern Germany. Microorganisms 9:1266. doi:10.3390/microorganisms9061266 34200876 PMC8230558

[56] Piesman J, Mather TN, Telford SR 3rd, Spielman A. 1986. Concurrent Borrelia burgdorferi and Babesia microti infection in nymphal Ixodes dammini. J Clin Microbiol 24:446–447. doi:10.1128/jcm.24.3.446-447.1986 3760136 PMC268931

[57] Vannier E, Richer LM, Dinh DM, Brisson D, Ostfeld RS, Gomes-Solecki M. 2023. Deployment of a reservoir-targeted vaccine against Borrelia burgdorferi reduces the prevalence of Babesia microti coinfection in Ixodes scapularis ticks. J Infect Dis 227:1127–1131. doi:10.1093/infdis/jiac462 36416014 PMC10175066

[58] Krause PJ, McKay K, Thompson CA, Sikand VK, Lentz R, Lepore T, Closter L, Christianson D, Telford SR, Persing D, Radolf JD, Spielman A, Deer-Associated Infection Study Group. 2002. Disease-specific diagnosis of coinfecting tickborne zoonoses: babesiosis, human granulocytic ehrlichiosis, and Lyme disease. Clin Infect Dis 34:1184–1191. doi:10.1086/339813 11941544

[59] Dugat T, Lagrée AC, Maillard R, Boulouis HJ, Haddad N. 2015. Opening the black box of Anaplasma phagocytophilum diversity: current situation and future perspectives. Front Cell Infect Microbiol 5:61. doi:10.3389/fcimb.2015.00061 26322277 PMC4536383

[60] Leiby DA. 2011. Transfusion-transmitted Babesia spp.: bull’s-eye on Babesia microti. Clin Microbiol Rev 24:14–28. doi:10.1128/CMR.00022-10 21233506 PMC3021205

[61] Tonnetti L, Townsend RL, Deisting BM, Haynes JM, Dodd RY, Stramer SL. 2019. The impact of Babesia microti blood donation screening. Transfusion 59:593–600. doi:10.1111/trf.15043 30499595

[62] Moritz ED, Winton CS, Tonnetti L, Townsend RL, Berardi VP, Hewins M-E, Weeks KE, Dodd RY, Stramer SL. 2016. Screening for Babesia microti in the U.S. blood supply. N Engl J Med 375:2236–2245. doi:10.1056/NEJMoa1600897 27959685

[63] Swanson M, Pickrel A, Williamson J, Montgomery S. 2023. Trends in reported babesiosis cases - United States, 2011-2019. Am J Transplant 23:582–584. doi:10.1016/j.ajt.2023.03.013 37024154

[64] Johnson TL, Graham CB, Maes SE, Hojgaard A, Fleshman A, Boegler KA, Delory MJ, Slater KS, Karpathy SE, Bjork JK, Neitzel DF, Schiffman EK, Eisen RJ. 2018. Prevalence and distribution of seven human pathogens in host-seeking Ixodes scapularis (Acari: Ixodidae) nymphs in Minnesota, USA. Ticks Tick Borne Dis 9:1499–1507. doi:10.1016/j.ttbdis.2018.07.009 30055987 PMC6594169

[65] Diuk-Wasser MA, Gatewood AG, Cortinas MR, Yaremych-Hamer S, Tsao J, Kitron U, Hickling G, Brownstein JS, Walker E, Piesman J, Fish D. 2006. Spatiotemporal patterns of host-seeking Ixodes scapularis nymphs (Acari: Ixodidae) in the United States. J Med Entomol 43:166–176. doi:10.1603/0022-2585(2006)043[0166:spohis]2.0.co;2 16619595

[66] Ostfeld RS, Canham CD, Oggenfuss K, Winchcombe RJ, Keesing F. 2006. Climate, deer, rodents, and acorns as determinants of variation in lyme-disease risk. PLoS Biol 4:e145. doi:10.1371/journal.pbio.0040145 16669698 PMC1457019

[67] Eisen RJ, Eisen L. 2018. The blacklegged tick, Ixodes scapularis: an increasing public health concern. Trends Parasitol 34:295–309. doi:10.1016/j.pt.2017.12.006 29336985 PMC5879012

[68] Kugeler KJ, Schwartz AM, Delorey MJ, Mead PS, Hinckley AF. 2021. Estimating the frequency of lyme disease diagnoses, United States, 2010-2018. Emerg Infect Dis 27:616–619. doi:10.3201/eid2702.202731 33496229 PMC7853543

[69] Gatewood AG, Liebman KA, Vourc’h G, Bunikis J, Hamer SA, Cortinas R, Melton F, Cislo P, Kitron U, Tsao J, Barbour AG, Fish D, Diuk-Wasser MA. 2009. Climate and tick seasonality are predictors of Borrelia burgdorferi genotype distribution. Appl Environ Microbiol 75:2476–2483. doi:10.1128/AEM.02633-08 19251900 PMC2675205

