# Supplementary material for: Coinfection ecology and pathogen emergence in a Borrelia-endemic landscape: 5 years of Borrelia burgdorferi, Anaplasma phagocytophilum, and Babesia microti surveillance in Maryland
Source: Appl Environ Microbiol. 2026 Apr 20;92(5):e02242-25. doi: 10.1128/aem.02242-25 (PMC13165503; doi:10.1128/aem.02242-25)
Supplement: Supplemental material — Tables S1 to S3; Fig. S1. [file aem.02242-25-s0001.docx]

# **Coinfection Ecology and Pathogen Emergence in a *Borrelia*-Endemic Landscape: Five Years of *Borrelia burgdorferi, Anaplasma phagocytophilum*, and *Babesia microti* Surveillance in Maryland**

Greg Joyner^1,2^, Olifan Abil^1^, Maria J. Sanches^2^, Amy Schwartz^3^, Julia Poje^3^, Kathryn Arnold^3^, Christine Petersen^3+^, Maria Gomes Solecki^1,2,^*

# Affiliations

1. Department of Microbiology, Immunology and Biochemistry, University of

Tennessee Health Science Center, Memphis, Tennessee, USA

1. Immuno Technologies Inc, Memphis, Tennessee, USA
2. University of Iowa, Iowa City, IA, USA

**SUPPLEMENTAL MATERIAL**

**Supplemental Table 1. Logistic regression of infection prevalence by year.** Logistic regression results testing for monotonic changes in infection prevalence over time (2020–2024), stratified by pathogen, tick type, and site. OR, odds ratio; CI, confidence interval.

| Pathogen | Tick source | Site | n tested | n positive | OR per year | 95% CI | p-value |
| --- | --- | --- | --- | --- | --- | --- | --- |
| *Anaplasma phagocytophilum* | All ticks | Statewide | 557 | 21 | 1.12 | 0.79–1.59 | 0.5369 |
| *Anaplasma phagocytophilum* | All ticks | Harford County | 165 | 8 | 1.39 | 0.71–2.70 | 0.3362 |
| *Anaplasma phagocytophilum* | All ticks | Montgomery County | 225 | 7 | 0.81 | 0.49–1.35 | 0.4191 |
| *Anaplasma phagocytophilum* | All ticks | Baltimore County | 167 | 6 | 1.84 | 0.67–5.03 | 0.236 |
| *Babesia microti* | All ticks | Statewide | 557 | 55 | 1.33 | 1.04–1.69 | 0.0221 |
| *Babesia microti* | All ticks | Harford County | 165 | 14 | 1.52 | 0.98–2.36 | 0.0637 |
| *Babesia microti* | All ticks | Montgomery County | 225 | 12 | 1.07 | 0.71–1.61 | 0.7395 |
| *Babesia microti* | All ticks | Baltimore County | 167 | 29 | 1.35 | 0.91–2.01 | 0.1326 |
| *Anaplasma phagocytophilum* | Drag only | Statewide | 333 | 9 | 1.42 | 0.86–2.35 | 0.171 |
| *Babesia microti* | Drag only | Statewide | 333 | 11 | 1.58 | 1.01–2.49 | 0.0462 |
| *Anaplasma phagocytophilum* | *Peromyscus leucopus*-fed | Statewide | 224 | 12 | 1.02 | 0.67–1.57 | 0.9154 |
| *Babesia microti* | *Peromyscus leucopus*-fed | Statewide | 224 | 44 | 1.21 | 0.95–1.54 | 0.1142 |
| *Anaplasma phagocytophilum* | *Peromyscus leucopus*-fed | Harford County | 54 | 6 | 1.49 | 0.83–2.69 | 0.176 |
| *Anaplasma phagocytophilum* | *Peromyscus leucopus*-fed | Montgomery County | 112 | 4 | 0.69 | 0.35–1.34 | 0.2655 |
| *Anaplasma phagocytophilum* | *Peromyscus leucopus*-fed | Baltimore County | 58 | 2 | 1.68 | 0.50–5.63 | 0.4017 |
| *Babesia microti* | *Peromyscus leucopus*-fed | Harford County | 54 | 8 | 1.73 | 1.05–2.86 | 0.0316 |
| *Babesia microti* | *Peromyscus leucopus*-fed | Montgomery County | 112 | 4 | 1.06 | 0.66–1.72 | 0.8086 |

**Supplemental Table 2. Confirmed human cases of Lyme disease, anaplasmosis, and babesiosis in Maryland by county, 2019–2023.** Case counts were obtained from CDC and Maryland Department of Health public surveillance data.

|  | Anaplasmosis | |  |  |  | Babesiosis |  |  |  |  | Lyme disease | |  |  |  |
| --- | --- | --- | --- | --- | --- | --- | --- | --- | --- | --- | --- | --- | --- | --- | --- |
| County | 2019 | 2020 | 2021 | 2022 | 2023 | 2019 | 2020 | 2021 | 2022 | 2023 | 2019 | 2020 | 2021 | 2022 | 2023 |
| Allegany | 0 | 0 | 0 | 0 | 0 | 0 | 0 | 0 | 0 | 0 | 70 | 51 | 84 | 109 | 153 |
| Anne Arundel | 0 | 1 | 3 | 1 | 1 | 0 | 0 | 0 | 0 | 3 | 110 | 70 | 153 | 127 | 162 |
| Baltimore City | 5 | 0 | 0 | 1 | 4 | 0 | 1 | 1 | 0 | 2 | 138 | 52 | 81 | 278 | 414 |
| Baltimore County | 2 | 0 | 0 | 0 | 0 | 2 | 2 | 2 | 2 | 0 | 41 | 13 | 24 | 100 | 54 |
| Calvert | 1 | 0 | 0 | 2 | 0 | 0 | 0 | 0 | 0 | 0 | 49 | 26 | 30 | 24 | 52 |
| Caroline | 0 | 0 | 0 | 0 | 0 | 0 | 0 | 0 | 0 | 0 | 12 | 10 | 7 | 6 | 16 |
| Carroll | 1 | 0 | 2 | 1 | 2 | 1 | 0 | 2 | 1 | 2 | 169 | 155 | 87 | 193 | 228 |
| Cecil | 1 | 1 | 0 | 0 | 1 | 0 | 0 | 1 | 0 | 0 | 61 | 35 | 44 | 61 | 104 |
| Charles | 0 | 0 | 0 | 0 | 0 | 0 | 0 | 0 | 0 | 0 | 12 | 9 | 8 | 29 | 42 |
| Dorchester | 0 | 0 | 0 | 0 | 2 | 0 | 0 | 0 | 0 | 0 | 4 | 2 | 4 | 9 | 9 |
| Frederick | 1 | 0 | 1 | 0 | 1 | 0 | 0 | 2 | 0 | 2 | 126 | 98 | 108 | 181 | 220 |
| Garrett | 0 | 0 | 0 | 1 | 2 | 0 | 0 | 0 | 0 | 0 | 26 | 16 | 37 | 84 | 87 |
| Harford | 0 | 0 | 0 | 1 | 4 | 1 | 0 | 0 | 0 | 2 | 140 | 85 | 84 | 94 | 147 |
| Howard | 1 | 0 | 2 | 2 | 0 | 1 | 1 | 1 | 0 | 3 | 115 | 89 | 46 | 166 | 211 |
| Kent | 0 | 0 | 0 | 0 | 0 | 0 | 0 | 0 | 0 | 0 | 20 | 8 | 1 | 14 | 27 |
| Montgomery | 2 | 4 | 6 | 6 | 3 | 1 | 3 | 3 | 2 | 8 | 134 | 22 | 14 | 328 | 125 |
| Prince George’s | 0 | 0 | 0 | 1 | 2 | 0 | 1 | 0 | 0 | 5 | 22 | 0 | 0 | 49 | 61 |
| Queen Anne’s | 0 | 0 | 0 | 0 | 0 | 0 | 0 | 0 | 0 | 0 | 44 | 25 | 25 | 22 | 40 |
| St. Mary’s | 0 | 0 | 0 | 0 | 0 | 0 | 0 | 0 | 0 | 0 | 37 | 1 | 5 | 33 | 65 |
| Somerset | 0 | 0 | 0 | 1 | 0 | 0 | 0 | 0 | 0 | 0 | 6 | 4 | 5 | 5 | 10 |
| Talbot | 0 | 0 | 0 | 0 | 1 | 0 | 0 | 0 | 0 | 1 | 19 | 6 | 5 | 21 | 11 |
| Washington | 1 | 0 | 0 | 0 | 1 | 0 | 0 | 0 | 0 | 0 | 36 | 41 | 32 | 0 | 107 |
| Wicomico | 1 | 0 | 1 | 0 | 2 | 0 | 0 | 0 | 1 | 0 | 12 | 15 | 7 | 5 | 29 |
| Worcester | 0 | 0 | 2 | 0 | 0 | 0 | 1 | 1 | 1 | 1 | 14 | 7 | 17 | 22 | 30 |

**Supplemental Table 3. Oligonucleotides used for primary detection qPCR and confirmatory PCR**

| **Pathogen** | **Assay purpose** | **Target** | **Oligo name** | **Sequence (5′→3′)** |
| --- | --- | --- | --- | --- |
| *Borrelia burgdorferi* | Primary detection | *flaB* | flaB-F | AAGCAATCTAGGTCTCAAGC |
| *Borrelia burgdorferi* | Primary detection | *flaB* | flaB-R | GCTTCAGCCTGGCCATAAATAG |
| *Borrelia burgdorferi* | Primary detection | *flaB* | flaB-Probe | FAM-AGATGTGGTAGACCCGAAGCCGAG-TAMRA |
| *Borrelia burgdorferi* | Confirmatory | *rrf–rrl* (5S–23S) IGS | IGS-F | CTGCGAGITCGCGGGAGA |
| *Borrelia burgdorferi* | Confirmatory | *rrf–rrl* (5S–23S) IGS | IGS-R | TCCTAGGCATTCACCATA |
| *Anaplasma phagocytophilum* | Primary detection | 16S rRNA | Ap-16S-F | CGGAATTCCTAGTGTAGAGGTGAAA |
| *Anaplasma phagocytophilum* | Primary detection | 16S rRNA | Ap-16S-R | GTCAGTACCGGACCAGATAGC |
| *Anaplasma phagocytophilum* | Primary detection | 16S rRNA | Ap-16S-Probe | VIC-CCACTGGTGTTCCTCC-MGB |
| *Anaplasma phagocytophilum* | Confirmatory | *groEL* (hsp60) | groEL-569F | ATGGTATGCAGTTTGATCGC |
| *Anaplasma phagocytophilum* | Confirmatory | *groEL* (hsp60) | groEL-1193R | TCTACTCTGTCTTTGCGTTC |
| *Anaplasma phagocytophilum* | Confirmatory | *groEL* (hsp60) | groEL-1142R | TTGAGTACAGCAACACCACCGGAA |
| *Babesia microti* | Primary detection | 18S rRNA | Bm-18S-F | GCATGGAATAATGAAGTAGGACTTTGGT |
| *Babesia microti* | Primary detection | 18S rRNA | Bm-18S-R | CCCCAACTGCTCCTATTAACCATT |
| *Babesia microti* | Primary detection | 18S rRNA | Bm-18S-Probe | FAM-CTCTGGCTCAATAACC-MGB |
| *Babesia microti* | Confirmatory | β-tubulin | F34 | TGTGGTAACCAGAT(t/c)GG(a/t)GCCAA |
| *Babesia microti* | Confirmatory | β-tubulin | R323 | TCnGT(a/g)TA(a/g)TGnCC(t/c)TT(a/g)GCCCA |
| *Babesia microti* | Confirmatory | β-tubulin | F79 | GA(a/g)CA(t/c)GGnATnGA(t/c)CCnGTAA |
| *Babesia microti* | Confirmatory | β-tubulin | R206 | AC(a/t/g)GA(a/g)TCCATGGT(a/t/g)CCnGG(t/c)T |

**
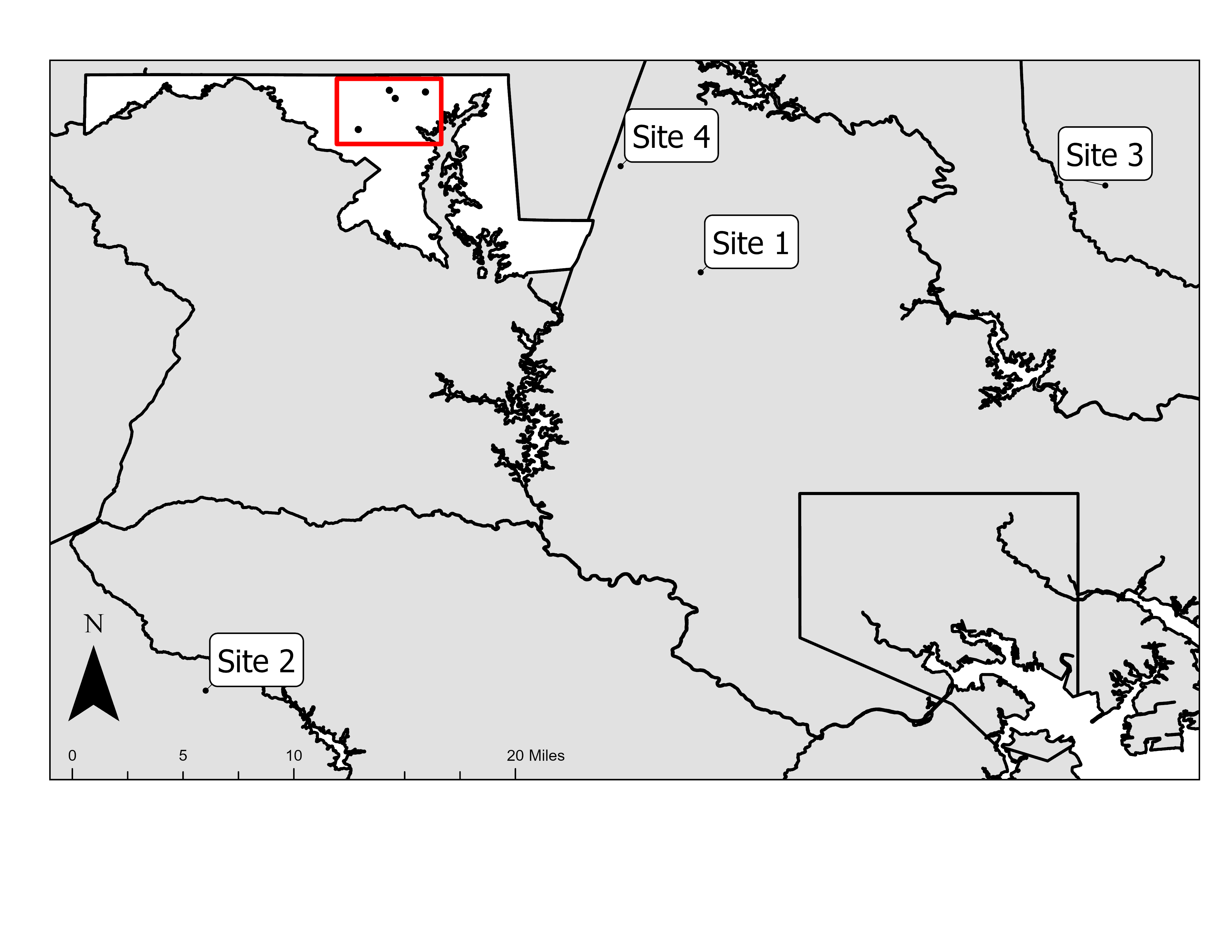
**

**Supplemental Figure 1.** Locations of field sites (Site 1 to Site 4) in the Mid-Atlantic state of Maryland. Sites were located in Harford, Baltimore, Montgomery, and Howard Counties.
