# Supplementary material for: Coinfection ecology and pathogen emergence in a Borrelia-endemic landscape: 5 years of Borrelia burgdorferi, Anaplasma phagocytophilum, and Babesia microti surveillance in Maryland
Source: Appl Environ Microbiol. Author manuscript; Available in PMC 2026 May 13. (PMC13165503; doi:10.1128/aem.02242-25)
Supplement: Supplementary file 1 [file NIHMS2168512-supplement-Joyner_AEM_Supplemental.pdf]

Coinfection Ecology and Pathogen Emergence in a *Borrelia*-Endemic Landscape: Five Years of *Borrelia burgdorferi, Anaplasma phagocytophilum*, and *Babesia microti* Surveillance in Maryland

Greg Joyner[1,2], Olifan Abil[1], Maria J. Sanches[2], Amy Schwartz[3], Julia Poje[3], Kathryn Arnold[3], Christine Petersen[3+], Maria Gomes Solecki[1,2,*]

Affiliations

1. Department of Microbiology, Immunology and Biochemistry, University of Tennessee Health Science Center, Memphis, Tennessee, USA
2. Immuno Technologies Inc, Memphis, Tennessee, USA
3. University of Iowa, Iowa City, IA, USA

SUPPLEMENTAL MATERIAL

Supplemental Table 1. Logistic regression of infection prevalence by year. Logistic regression results testing for monotonic changes in infection prevalence over time (2020–2024), stratified by pathogen, tick type, and site. OR, odds ratio; CI, confidence interval.

Pathogen	Tick source	Site	n tested	n positive	OR per year	95% CI	p-value
Anaplasma phagocytophilum	All ticks	Statewide	557	21	1.12	0.79–1.59	0.5369
Anaplasma phagocytophilum	All ticks	Harford County	165	8	1.39	0.71–2.70	0.3362
Anaplasma phagocytophilum	All ticks	Montgomery County	225	7	0.81	0.49–1.35	0.4191
Anaplasma phagocytophilum	All ticks	Baltimore County	167	6	1.84	0.67–5.03	0.236
Babesia microti	All ticks	Statewide	557	55	1.33	1.04–1.69	0.0221
Babesia microti	All ticks	Harford County	165	14	1.52	0.98–2.36	0.0637
Babesia microti	All ticks	Montgomery County	225	12	1.07	0.71–1.61	0.7395
Babesia microti	All ticks	Baltimore County	167	29	1.35	0.91–2.01	0.1326
Anaplasma phagocytophilum	Drag only	Statewide	333	9	1.42	0.86–2.35	0.171
Babesia microti	Drag only	Statewide	333	11	1.58	1.01–2.49	0.0462
Anaplasma phagocytophilum	*Peromyscus leucopus*-fed	Statewide	224	12	1.02	0.67–1.57	0.9154
Babesia microti	*Peromyscus leucopus*-fed	Statewide	224	44	1.21	0.95–1.54	0.1142
Anaplasma phagocytophilum	*Peromyscus leucopus*-fed	Harford County	54	6	1.49	0.83–2.69	0.176

Anaplasma phagocytophilum	*Peromyscus leucopus*-fed	Montgomery County	112	4	0.69	0.35–1.34	0.2655
Anaplasma phagocytophilum	*Peromyscus leucopus*-fed	Baltimore County	58	2	1.68	0.50–5.63	0.4017
Babesia microti	*Peromyscus leucopus*-fed	Harford County	54	8	1.73	1.05–2.86	0.0316
Babesia microti	*Peromyscus leucopus*-fed	Montgomery County	112	4	1.06	0.66–1.72	0.8086

Supplemental Table 2. Confirmed human cases of Lyme disease, anaplasmosis, and babesiosis in Maryland by county, 2019–2023. Case counts were obtained from CDC and Maryland Department of Health public surveillance data.

	Anaplasmosis					Babesiosis					Lyme disease				
County	2019	2020	2021	2022	2023	2019	2020	2021	2022	2023	2019	2020	2021	2022	2023
Allegany	0	0	0	0	0	0	0	0	0	0	70	51	84	109	153
Anne Arundel	0	1	3	1	1	0	0	0	0	3	110	70	153	127	162
Baltimore City	5	0	0	1	4	0	1	1	0	2	138	52	81	278	414
Baltimore County	2	0	0	0	0	2	2	2	2	0	41	13	24	100	54
Calvert	1	0	0	2	0	0	0	0	0	0	49	26	30	24	52
Caroline	0	0	0	0	0	0	0	0	0	0	12	10	7	6	16
Carroll	1	0	2	1	2	1	0	2	1	2	169	155	87	193	228
Cecil	1	1	0	0	1	0	0	1	0	0	61	35	44	61	104
Charles	0	0	0	0	0	0	0	0	0	0	12	9	8	29	42
Dorchester	0	0	0	0	2	0	0	0	0	0	4	2	4	9	9
Frederick	1	0	1	0	1	0	0	2	0	2	126	98	108	181	220
Garrett	0	0	0	1	2	0	0	0	0	0	26	16	37	84	87
Harford	0	0	0	1	4	1	0	0	0	2	140	85	84	94	147
Howard	1	0	2	2	0	1	1	1	0	3	115	89	46	166	211
Kent	0	0	0	0	0	0	0	0	0	0	20	8	1	14	27
Montgomery	2	4	6	6	3	1	3	3	2	8	134	22	14	328	125
Prince George’s	0	0	0	1	2	0	1	0	0	5	22	0	0	49	61
Queen Anne’s	0	0	0	0	0	0	0	0	0	0	44	25	25	22	40
St. Mary’s	0	0	0	0	0	0	0	0	0	0	37	1	5	33	65
Somerset	0	0	0	1	0	0	0	0	0	0	6	4	5	5	10
Talbot	0	0	0	0	1	0	0	0	0	1	19	6	5	21	11
Washington	1	0	0	0	1	0	0	0	0	0	36	41	32	0	107

Wicomico	1	0	1	0	2	0	0	0	1	0	12	15	7	5	29
Worcester	0	0	2	0	0	0	1	1	1	1	14	7	17	22	30

Supplemental Table 3. Oligonucleotides used for primary detection qPCR and confirmatory PCR

Pathogen	Assay purpose	Target	Oligo name	Sequence (5′→3′)
Borrelia burgdorferi	Primary detection	*flaB*	flaB-F	AAGCAATCTAGGTCTCAAGC
Borrelia burgdorferi	Primary detection	*flaB*	flaB-R	GCTTCAGCCTGGCCATAAATAG
Borrelia burgdorferi	Primary detection	*flaB*	flaB-Probe	FAM-AGATGTGGTAGACCCGAAGCCGAG-TAMRA
Borrelia burgdorferi	Confirmatory	*rrf–rrl* (5S–23S) IGS	IGS-F	CTGCGAGITCGCGGGAGA
Borrelia burgdorferi	Confirmatory	*rrf–rrl* (5S–23S) IGS	IGS-R	TCCTAGGCATTCACCATA
Anaplasma phagocytophilum	Primary detection	16S rRNA	Ap-16S-F	CGGAATTCCTAGTGTAGAGGTGAAA
Anaplasma phagocytophilum	Primary detection	16S rRNA	Ap-16S-R	GTCAGTACCGGACCAGATAGC
Anaplasma phagocytophilum	Primary detection	16S rRNA	Ap-16S-Probe	VIC-CCACTGGTGTTCCTCC-MGB
Anaplasma phagocytophilum	Confirmatory	*groEL* (hsp60)	groEL-569F	ATGGTATGCAGTTTGATCGC
Anaplasma phagocytophilum	Confirmatory	*groEL* (hsp60)	groEL-1193R	TCTACTCTGTCTTTGCGTTC
Anaplasma phagocytophilum	Confirmatory	*groEL* (hsp60)	groEL-1142R	TTGAGTACAGCAACACCACCGGAA
Babesia microti	Primary detection	18S rRNA	Bm-18S-F	GCATGGAATAATGAAGTAGGACTTTGGT
Babesia microti	Primary detection	18S rRNA	Bm-18S-R	CCCCAACTGCTCCTATTAACCATT
Babesia microti	Primary detection	18S rRNA	Bm-18S-Probe	FAM-CTCTGGCTCAATAACC-MGB
Babesia microti	Confirmatory	β-tubulin	F34	TGTGGTAACCAGAT(t/c)GG(a/t)GCCAA
Babesia microti	Confirmatory	β-tubulin	R323	TCnGT(a/g)TA(a/g)TGnCC(t/c)TT(a/g)GCCCA
Babesia microti	Confirmatory	β-tubulin	F79	GA(a/g)CA(t/c)GGnATnGA(t/c)CCnGTAA
Babesia microti	Confirmatory	β-tubulin	R206	AC(a/t/g)GA(a/g)TCCATGGT(a/t/g)CCnGG(t/c)T

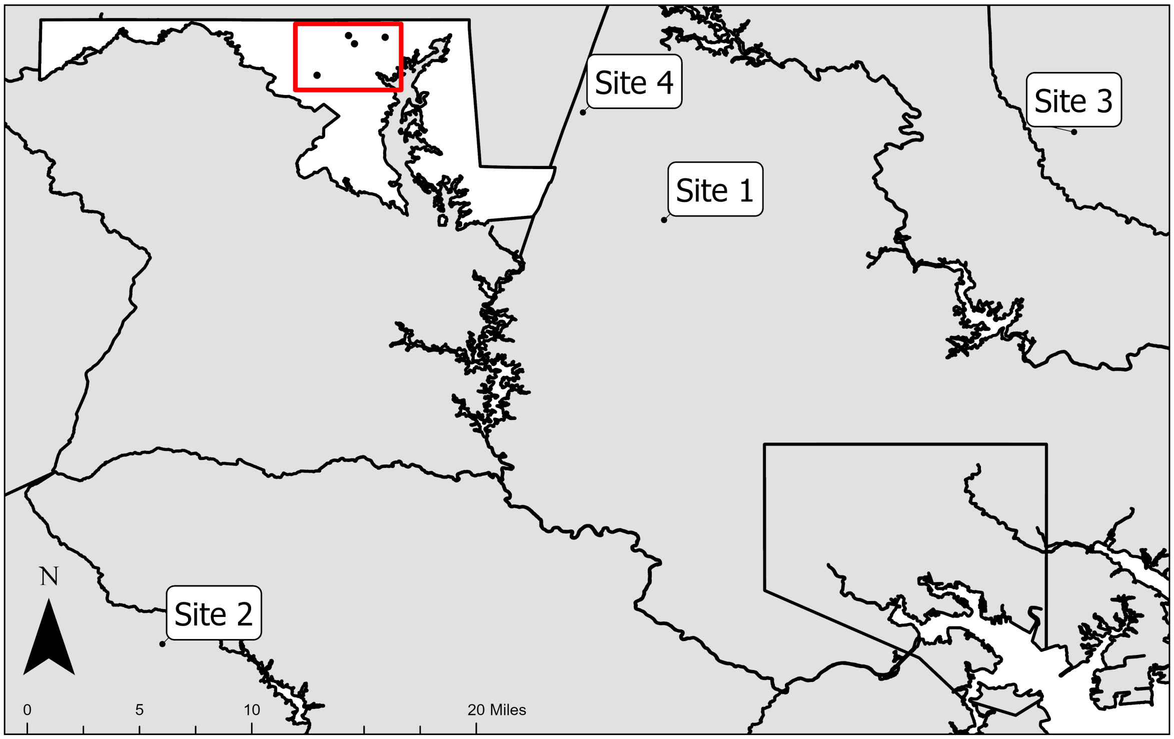

Supplemental Figure 1. Locations of field sites (Site 1 to Site 4) in the Mid-Atlantic state of Maryland. Sites were located in Harford, Baltimore, Montgomery, and Howard Counties.
